# Supplementary material for: Distribution of Prophages in the Oenococcus oeni Species
Source: Microorganisms. 2021 Apr 16;9(4):856. doi: 10.3390/microorganisms9040856 (PMC8074189; doi:10.3390/microorganisms9040856)
Supplement: Supplementary file 1 [file microorganisms-09-00856-s001.zip › microorganisms-1175108-supplementary.pdf]

Table S1 Genomes of lysogenic and non-lysogenic strains of ***O. oeni*** used in this study.

Strain characteristics	Genome accession number
Lysogens (134)	ACSE01, AJIJ01, AJTO01, ALAD01, ALAE01, ALAG01, ALAH01, ALAJ01, ALAK01, AZHH01, AZIP01, AZJU01, AZJV01, AZJW01, AZJX01, AZJY01, AZKA01, AZKB01, AZKC01, AZKE01, AZKF01, AZKH01, AZKJ01, AZKL01, AZLG01, AZLK01, AZLN01, AZLP01, CP014324, CP027431, CP038451 , JPEK01, JPEM01, LKRT01, LKRU01, LKRV01, LKRZ01, LKSA01, LKSB01, LKSC01, LKSD01, LKSE01, LKSR01, MLKP01, MLKQ01, MLKT01, MLKZ01, MLLA01, MLLB01, MLLD01, MLLE01, MLLG01, MLLK01, MLLM01, MLLN01, MLLO01, MLLQ01, MLLS01, MLLU01, MLLX01, MLLZ01, MLMF01, MLMI01, MLMJ01, MLMK01, MLMP01, MLMT01, MLMW01, MLMY01, MLMZ01, MLNA01, MLNC01, MLND01, MLNE01, MLNH01, MLNK01, MLNL01, MLNN01, MLNP01, MLNQ01, MLNS01, MLNU01, MLNV01, MLNX01, MLNY01, MLNZ01, MLOA01, MLOB01, MLOC01, MLOE01, MLOF01, MLOG01, MLOH01, MLOI01, MLOJ01, MLOL01, MLON01, MLOO01, MLOP01, MLOU01, MLOW01, MLOX01, MLOY01, MLPB01, MLPC01, MLPD01, MLPE01, MLPG01, MLPH01, MLPI01, MLPK01, MLPL01, MLPN01, MLPQ01, MLPR01, MLPS01, MLPT01, MLPV01, MLPW01, MLPX01, MLPY01, SNST01, SNSU01, SNSV01, SNSW01, SNSX01, SNSY01, ULFS01, ULFU01, ULFZ01, ULGA01, ULGB01, ULGC01, ULGD01.
Non-lysogenic strains (97)	AAUV01, ALAF01, ALAI01, AZJZ01, AZKD01, AZKG01, AZKI01, AZKK01, AZLE01, AZLF01, AZLH01, AZLI01, AZLJ01, AZLL01, AZLM01, AZLO01, AZLQ01, AZLR01, AZLS01, AZLT01, CP008528, JMIS01, JOOH01, JPEJ01, JPEL01, LKRS01, LKRW01, LKRX01, LKRY01, LOBV01, LR031358, MDWO01, MEHP01, MLKR01, MLKS01, MLKU01, MLKV01, MLKW01, MLKX01, MLKY01, MLLC01, MLLF01, MLLH01, MLLI01, MLLJ01, MLLL01, MLLP01, MLLR01, MLLT01, MLLV01, MLLW01, MLLY01, MLMA01, MLMB01, MLMC01, MLMD01, MLME01, MLMG01, MLMH01, MLML01, MLMN01, MLMO01, MLMQ01, MLMR01, MLMS01, MLMU01, MLMV01, MLMX01, MLNB01, MLNF01, MLNG01, MLNI01, MLNJ01, MLNM01, MLNO01, MLNR01, MLNT01, MLNW01, MLOD01, MLOK01, MLOM01, MLOQ01, MLOR01, MLOS01, MLOV01, MLOZ01, MLPA01, MLPF01, MLPJ01, MLPM01, MLPO01, MLPP01, QYTB01, ULFT01, ULFV01, ULFW01, ULFX01 .

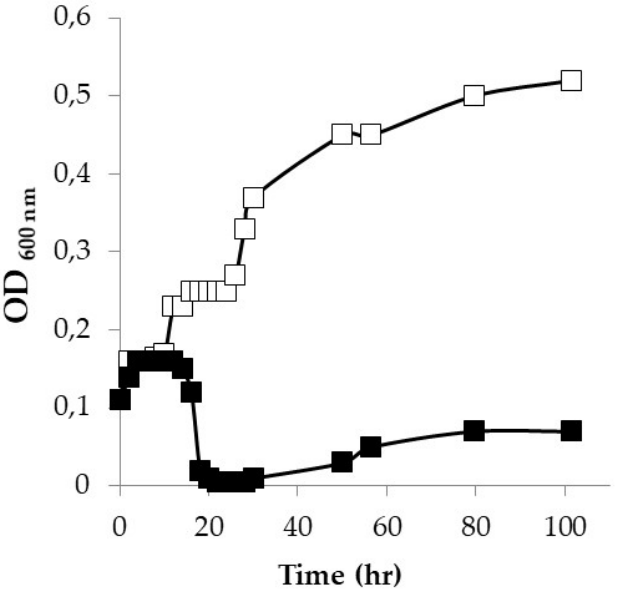

Figure S1 Kinetics of bacteriophage induction from the lysogen *O. oeni* IOEB0608 with (■) and without (□) mitomycin C treatment (1 µg/mL). Each culture was grown in MRS broth to an OD_{600} of 0.2-0.3 and then separated into two aliquots, one of which was induced with mitomycin C.

```
IntF      MATIAYQSKTKTYRVQIITPDHKRVGKSGFHTKTAAKTWLTENQLKIVTGKSEVNSSHEL    60
IntB-F    MASITYQEKVKTYRVQVTTKGHKRVGKSGFRTKTEARKWLTENELKIMTGKSDLIDSSKL    60

IntF      LSDYFKHWYETYKTNVTDITLDQYKTTYRIIEKYLPHVRLNDFTREKYQKFLNKYGEDHA    120
IntB-F    LSSYFEHWYETYKTNVTDITLDQYKTTYRIIKKYLPHARLNNFTREQYQKFLNKYGKDHA    120

IntF      KETVAKRKTHISACLKDAFADKLISEDITQRLTLTGKAGKSSELKFLEYEDFKNLEQYSY    180
IntB-F    KETVAKRKTHISACLKDAFADKLISEDITQRITLTGKAGKSSELKFLEADDFKRLEQYSY    180

IntF      DHLNNDSQLAIFIAIHTGMRIGEIRALKIKNVDFVHSKITIDKAMDGYGNIKAPKTVASN    240
IntB-F    DHLNNDSQLAIFIAIHTGMRIGEIRALKIKNVDFVQSKITIDKAMDGYGKIKAPKTAASN    240

IntF      RVIKIDKRLLDVLKGYKRVSGLLVQVTREAINHVLTKDLKHIGAKNVTFHALRHSHASYL    300
IntB-F    RVIQIDKRLLDVLKRYKRVSGLLVQVTREAINHVLTRDLKKIEAKDVTFHALRHSHASYL    300

IntF      LSKGVSIQYVSERLGHSSVAITENVYSHLLQTLRENEENKVTDLMNFQ                348
IntB-F    LSKGVSIQYVSERLGHSNVGITENVYSHLLKTLRENEEKKITDLMDFQ                348
```

Figure S2 Comparisons of the IntF and IntB protein sequences**.** Mutations are in grey. The four conserved amino acids in the C-terminal region (catalytic domain) of all integrases found in *O. oeni* are in red.

```
IntF      ATGGCAACAATTGCTTATCAATCCAAAACAAAAACATACCGTGTTC---------AGATC    51
PRF       ATGGCAACCTATATGAAACGTGGTTCAACTTGGCAAGCACGTGTATCA------------    48
IntD      ATGGCTTCAATTAAAAAACGCGGAAAATATTATCAAGCACGAGTATCCTACAGGGATCCA    60
          *****  *   *    * *       *        *   ** **

IntF      ATTACTCCAGATCATAAGCGTGTTGGCAAGTCGGGTTTTCATACAAAAACAGCAGCCAAA    111
PRF       ------AAGGATAAGCATCGATTCAATAAATCTGGTTTTGCTACCAAAAGAGAAGCAATT    102
IntD      GTTACCGGAGAATTCCGGACCAAAAATAAAAGTGGTTTTCTTAAAACAAAAGATGCACAA    120
                   **                ** ** ******  **  * ** **  **

IntF      ACATGGCTAACCGAAAACCAGCTTAAAATTGTTACCGGCAAATCAGAAGTAAATAGTTCA    171
PRF       ATTTGGGCTTCTAAAATAG---------AATTAGGCGAAGAG---AATAAACCAAAAAAT    150
IntD      ATCTGGGTTGGCCAAGTAT---------TATCTGGGAAACAACAAGAAAAAGAACAGCCT    171
          *  ***       **                         *     *   *

IntF      CACGAATTATTGTCTGATTATTTTAAGCATTGGTATGAAACATATAAAACAAATGTAACG    231
PRF       AATATTTTATTTTCTGATTATTTTAAAAAATGGTATGAGACATATAAAACAAATCGGACC    210
IntD      GACATACTTTTATCTGATTACTTTGAGAATTGGTACCTCACATATCGTGTCAACCGATCA    231
           *     * ** ******** *** *  * *****    ******      **     *

IntF      GATATAACGCTCGACCAGTACAAAACGACTTATCGTATTATTGAAAAATATTTACCACAT    291
PRF       GATGTCACTTTATTACAGTACAAAAACACTGACTATGTAATTGATAAATATTTAAAGGGT    270
IntD      AATCAAACTATATACCAGTATCGAGACACACTTCACACAATTAAGAAGTATTTGCCCACG    291
           **   **       *****       *            **   ** *****

IntF      GTGCGACTGAATGATTTTACCCGTGAGAAATATCAGAAATTTTTAAACAAATATGGCGAA    351
PRF       GAAATTTTAAATAACTTAACTCGGGCTAAATTACAACAATTTATCAATGAATACGGCAAA    330
IntD      ATGACTTTGAAATCGTTCACACGATCTAATTTTCAGACATTTATTAATAATTTTGGTAAA    351
                 * **    ** ** **    ** *  **   **** * **  * *  **  **

IntF      GATCACGCTAAAGAAACTGTTGCTAAACGAAAAACACACATCTCAGCTTGTTTAAAAGAC    411
PRF       GACCACGCTAAAGAGACTGTCCAAAAACATAAGGGCATATTATCGCCTG-CTTAAAAGAT    389
IntD      GATCACGCTGAAAAAACAGTACAAAAACGCAAGGGTCAAATTTCGGCCGCTTTAAAAGAT    411
          ** ****** ** * ** **    ****  **        * **  *    ********

IntF      GCTTTTGCCGATAAATTAATCAGTGAAGACATAACACAACGATTGACCCTAACTGGTAAA    471
PRF       GCTTATCATGAAGGGCTAATCAAACAAGACGTTACTTATAGATTGAATCTAGTCTATAAC    449
IntD      GCCTTAGCTGAAGAATTGATAATTAAGGATCCTACTATAAGAATTGGTTTAGTGTACAAT    471
          ** *     **     * ** *   * **    **     ** *     **      **

IntF      GCCGGCA------AGTCGTCAGAGCTTAAATTCTTGGAGTATGAAGATTTTAAGAACTTA    525
PRF       CAAAAAACTATCAAACCAATCGAAGAAAAATTCCTAGAGGCAGAAGACGCACATAAACTC    509
IntD      AATGCAACTAAAGTGCCTATTGAAAATAAATATTTGGAATTGAATGAAGCTGAAAAATTG    531
          *         *    **    ****   * **     * **     * **  *

IntF      GAGCAATATTCATATGATCATCTCAATAACGATTCACAGCTGGCTATTTTTATAGCTATT    585
PRF       GTTGCTTATTGCGAAAAGAACATCACTA---GAGGCAACTTTTGTATTCTGACCGGTATT    566
IntD      ATAGACCATTGTATAAAGAACCTATCTA---GGGGTAATTTTATGATATTAACTGGTCTT    588
          ***      *  *  *   **         *  *    **  * *  * * **

IntF      CATACAGGCATGCGAATAGGTGAAATTAGAGCTTTAAAGATTAAAAATGTAGATTTTGTG    645
PRF       CTATCAGGAGCCAGATTTGGTGAAGTTAGAGCCTTGATTGACAGTGATATAGACACGAAG    626
IntD      CTGTCAGGAGCAAGATTTGGTGAGCTTCGAGCATTAACCGATGCAGATATCGATACCAAA    648
          *   ****     ** * *****  ** **** **  *         ** * **

IntF      CATTCTAAAATTACTATTGATAAAGCTATGGACGGTTATGGCAATATCA---AAGCACCA    702
PRF       AACCACACTATTAGTATTAACAAGGCTGTTGATAGGCTAACAGGAAAAGATAAGGAAACC    686
IntD      AAACATCTCATCTCTATTACTAAAGCAGTTGATAAATTTACCAATAAGGATAAAGCTCCA    708
          *       **   ****   ** **  * **             *      * *   *

IntF      AAAACGGTCGCTAGTAACCGTGTAATTAAAATCGATAAACGATTATTAGACGTATTAAAG    762
PRF       AAAAACAAGCAATCCACAAGAATTATAGCTATGCCCGACAGATGGTTTCAAATATATAAG    746
IntD      AAAACCAAGAATTCGATTAGAGAAATCATAATGCCGGATAAATGGTTTGAAGTTTATAGT    768
          ****           *   *    **    **     *   **  **  *  * *  *

IntF      GGATATAAGCGTGTTTCTGGTTTATTGGTACAAGTAACAAGAGAAGCAATTAACCACGTT    822
PRF       AATTACAACCATGATGGAAAACGGCTTTTTGACATCAGCTCAAACGCTATCAACAAAGAT    806
IntD      GATTTCAAGCATAATGACAAGCGACTTTTTGACATGACATCTAACGGAATCAACAAAGAC    828
          *  ** * *  *          *  *  *  * *      * *  ** *** * *

IntF      CTAACGAAAGATTTAAAGCACATAGGGGCAAAAAACGTGACCTTTCATGCTTTGCGTCAC    882
PRF       ATGAGATATATCGCAAAGAAAATCGATATAAAGCCTGTCACCTATCACGCTTTAAGGCAC    866
IntD      TTAAAATATTTATGTGAAAAACTAGAAATCAAGCACGTTACGTTTCACGCTTTAAGGCAT    888
          * *   *        *  *  * *     **    **  ** * *** *****  * **

IntF      AGCCATGCCAGCTATTTATTATCCAAAGGTGTTTCAATTCAATATGTATCCGAACGTTTG    942
PRF       ACTCACGCTTCAATGCTCTTGGCCAATAACGTTTCAATGCAATATGTTTCCGAAAGATTA    926
IntD      ACGCATGCTTCAATGCTATTAGCACATGATATTTCAATGCAATATGTGTCTGGAAGATTA    948
          *  ** **        * **  *  *     ******* ******** ** * * * **

IntF      GGGCATTCCAGTGTTGCTATCACAGAAAATGTTTACTCTCATCTACTTCAAACGTTACGG    1002
PRF       GGACACGCTAATCTATCAATCACGGAAAAAGTCTACTCTCACCTACTTGAAGATAAGAGA    986
IntD      GGACATGCCAATCTTTCGATAACAGAACAGGTATATTCGCATCTTTTAGAAGAAAAGAAA    1008
          ** **  * * * *  * ** ** *** * ** ** ** ** **  *  **

IntF      GAAAACGAAGAAAACAAGGTCACTGATTTAATGAATTTTCAGTGA          1047
PRF       AAAGAAGAAGAAAAAAAGGGCGATGGATATTTTTTAA---------          1022
IntD      AATCAAGAAGAAATGAAAGCAATGAAGATATTTTAG---------          1044
          *  * *******  *  *  *   *  *  *  *
```

Figure S3 Alignments of the nucleotide sequences of the integrase genes from PR_F and Int_F and Int_D prophages. The yellow color represents deletions in *intPR*$_F$ and *int*$_D$, sequences. The blue color represents deletions only observed in the *int*$_F$ sequence.
